# PANoptosis in urological diseases: molecular mechanisms, pathological roles, and emerging therapeutic opportunities

**DOI:** 10.3389/fimmu.2025.1729348

**Published:** 2026-01-09

**Authors:** Xin Di, Xin Jin, Shuqiang Feng, Ranwei Li

**Affiliations:** 1 Department of Respiratory and Critical Care Medicine, The Second Hospital of Jilin University, Changchun, Jilin, China; 2 Department of Oncology and Hematology, The Second Hospital of Jilin University, Changchun, Jilin, China; 3 Department of Urology, The Second Hospital of Jilin University, Changchun, Jilin, China

**Keywords:** biomarkers, cell death mechanisms, PANoptosis, therapeutic targeting, urological diseases

## Abstract

PANoptosis, a recently identified inflammatory programmed cell death pathway, integrates features of apoptosis, pyroptosis, and necroptosis and is regulated by a macromolecular complex called the PANoptosome. This pathway extends beyond conventional single-mode death mechanisms, marked a significant advancement in cell death research. Its emerging role in the pathogenesis and progression of urological diseases—including acute and chronic kidney injury, prostate cancer, renal carcinoma, and testicular pathologies—is increasingly recognized. This review outlines the molecular mechanisms and regulatory networks underlying PANoptosis and emphasizes its dual role, both in promoting tissue damage and in driving antitumor immunity. In addition, we discuss novel diagnostic biomarkers and therapeutic strategies targeting key components of PANoptosis, including multi-omics-based biomarker screening, nanomaterial-mediated delivery systems, and combination therapies. Advances in technologies, such as single-cell sequencing and spatial transcriptomics, are expanding precision medicine approaches, positioning PANoptosis as a promising target for improving clinical outcomes in urological diseases.

## Introduction

PANoptosis, a programmed inflammatory cell death pathway described in recent years, is defined by the simultaneous integration of molecular features from three death pathways: pyroptosis, apoptosis, and necroptosis. It cannot be fully explained by any single pathway (1). This process is regulated by the multiprotein PANoptosome complex and can be triggered by diverse upstream stimuli, including pathogen-associated molecular patterns (PAMPs), damage-associated molecular patterns (DAMPs), and cytokines (2–4). PANoptosis represents a tightly coordinated and inflammatory cell death mechanism that contributes to multiple pathological conditions, such as infectious diseases, cancer, autoimmune disorders, and metabolic diseases, underscoring extensive molecular crosstalk and compensatory mechanisms among programmed cell death pathways (5–7).

## Concept and molecular basis of PANoptosis

### Molecular composition of the PANoptosome: sensors

The sensor components of the PANoptosome are primarily pattern recognition receptors (PRRs) that detect diverse pathogenic or stress signals and initiate complex assembly (8). Identified sensors include Z-DNA binding protein 1 (ZBP1), receptor-interacting protein kinase 1 (RIPK1), and several inflammasome-associated receptors such as nucleotide-binding domain leucine-rich repeat and pyrin domain-containing receptor 3 (NLRP3), nucleotide-binding oligomerization domain-like receptor protein 4 (NLRC4), absent in melanoma 2 (AIM2), pyrin, nucleotide-binding oligomerization domain-like receptor protein 5 (NLRC5), and nucleotide-binding domain leucine-rich repeat and pyrin domain-containing receptor 12(NLRP12) (9–12).

ZBP1 is a dsDNA sensor that contains two nucleic acid–binding domains, Zα1 and Zα2, and a receptor-interacting protein homotypic interaction motif (RHIM) (13). The RHIM domain recruits RIPK1 and RIPK3, where the Zα2 domain is crucial for sensing influenza viruses (14, 15). ZBP1 recognizes left-handed Z-conformation nucleic acids (Z-RNA or Z-DNA) through its Zα2 domain (16). These conformational nucleic acids can arise from viral replication intermediates (such as influenza A virus [IAV]), mitochondria stress-released mitochondrial DNA (mtDNA), or DNA damage response products, thereby activating PANoptosome assembly in models of influenza infection, UV-induced damage, and sepsis (17–19). In lupus nephritis (LN), ZBP1 expression is upregulated in the kidneys, and ZBP1-mediated PANoptosis serves as a critical mechanism underlying renal injury (19). AIM2 is a cytoplasmic double-stranded DNA sensor that binds DNA through its HIN200 domain. In herpes simplex virus 1 (HSV-1) infection or cisplatin-induced acute kidney injury, AIM2 cooperates with ZBP1 to activate the caspase-8/GSDME axis (20, 21). In sepsis-induced acute kidney injury (AKI), AIM2 expression is significantly upregulated and targeted for activation by the EIF2AK2 protein, thereby driving PANoptosis in renal tubular epithelial cells (22). NLRP3, a multimodal inflammasome receptor, responds to K+ efflux, reactive oxygen species (ROS) bursts, and lysosomal rupture. Even in the absence of caspase-1 or gasdermin D (GSDMD), NLRP3 can promote PANoptosome assembly through the apoptosis-associated speck-like protein(ASC)–caspase-8–RIPK3 axis (23). In renal ischemia-reperfusion injury (IRI), the inhibitor 3,4-Methylenedioxy-β-Nitrostyrene(MNS) suppresses NLRP3, thereby reducing the formation of the PANoptosome and conferring renal protection (24).Noncanonical inflammasome sensors such as NLRP12 and NLRC5, can recruit caspase-8 and activate GSDMD in response to combined heme and PAMP stimulation or NAD+ depletion, driving PANoptosis (25–27).

### Molecular composition of the PANoptosome: adapters

Adapter proteins serve as central bridges within the PANoptosome, linking sensors to effectors and mediating signal transduction through specialized protein interaction domains. ASC is a critical adapter, with its N-terminal pyrin domain (PYD) bindings to the PYD of sensors such as AIM2 and NLRP3, whereas its C-terminal caspase activation and recruitment domain (CARD) recruits CARD-containing caspase-1 or caspase-8 (28, 29). This assembly forms a “supercomplex” in models of periodontitis and Alzheimer’s disease, markedly amplifying inflammatory signals (30, 31). In trichloroethylene (TCE)-induced immune-mediated kidney injury, multiplex immunofluorescence demonstrates the co-localization of ASC with proteins such as pMLKL and cleaved caspase-3, providing direct evidence of PANoptosome assembly (32).Fas-associated death domain protein (FADD) interacts with caspase-8 via its death effector domain (DED), and with RIPK1 through its death domain (DD), integrating death signals at the crossroads of extrinsic apoptosis and necroptosis (33). Additionally, the RHIM domain mediates strong interactions among ZBP1, RIPK1, and RIPK3, promoting amyloid-like fiber formation and thereby providing a scaffold for necroptotic signaling (34).

### Molecular composition of the PANoptosome: effectors

Effectors are the terminal executors of the PANoptosome, synergistically activating core execution molecules from apoptosis, pyroptosis, and necroptosis to drive irreversible inflammatory cell death. In apoptosis, caspase-8 acts as an initiator, by cleaving Bid to generate truncated Bid (tBid), which induces mitochondrial outer membrane permeabilization (MOMP) and cytochrome c release (35). This activates the caspase-9/caspase-3 cascade and directly engages the downstream effector caspases-3 and -7 to execute apoptosis. Activated caspase-3 cleaves GSDME, inducing pyroptosis-like lytic cell death (36). Pyroptosis is executed by inflammatory caspases (including caspase-1/4/5/11) which cleave GSDMD (37). The resulting N-terminal fragment (GSDMD-N) is inserted into the plasma membrane forming pores, that facilitate the maturation and release of IL-1β and IL-18. Under specific conditions, such as NLRP12 activation, caspase-8 can substitute for caspase-1 in cleaving GSDMD and simultaneously cleave GSDME, thereby intensifying lytic cell death (18, 25). Terminal necroptosis is triggered by RIPK3-mediated phosphorylation of MLKL. Phosphorylated MLKL disrupts plasma membrane integrity. RIPK1 exerts dual regulation in this process, either by promoting the RIPK3 activation or limiting excessive cell death through its scaffolding function (34, 38).

### Upstream and downstream regulatory mechanisms

The initiation and magnitude of PANoptosis are subject to precise multilevel regulation, with several upstream molecules acting as key molecular switches. Interferon regulatory factor 1 (IRF1) is a central transcriptional regulator under various stress conditions, synergistically activated by tumor necrosis factor-alpha (TNF-α) and interferon-gamma (IFN-γ) through the Janus Kinase/Signal Transducer and Activator of Transcription pathway (39). IRF1 upregulates multiple PANoptosome components, including ZBP1, RIPK1, and AIM2, driving PANoptosis in models of inflammatory bowel disease (IBD) and alcoholic liver disease (40–42). Transforming growth factor-β-activated kinase 1 (TAK1) maintains cellular homeostasis; its inactivation—for example, by the Yersinia effector protein YopJ—relieves RIPK1 inhibition, promoting RIPK1 phosphorylation and PANoptosome assembly, which induces strong inflammatory cell death (43, 44). Adenosine deaminase acting on RNA 1 (ADAR1), competes with ZBP1 for Z-RNA binding via its Zα domain, thereby inhibiting ZBP1 activation (45). ADAR1 loss-of-function or mutation results in the accumulation of endogenous Z-RNA, relieving ZBP1 inhibition and strongly driving ZBP1-dependent PANoptosis, which is critical for tumor immune editing and treatment resistance (46).

### Novel regulators: non-coding RNAs and metabolic reprogramming

Recent studies have highlighted non-coding RNAs and metabolic reprogramming as pivotal regulators of PANoptosis (47). Non-coding RNAs modulate PANoptosome components post-transcriptionally: circular RNA circOGDH binds to HMGB1 in diabetic cardiomyopathy models, stabilizing RIPK3 protein and promoting its activation, thereby exacerbating PANoptosis (48); long non-coding RNA AC133552.2 functions as a competitive endogenous RNA (ceRNA) in osteosarcoma, sequestering miR-454-3p to enhance IRF1 mRNA expression, and NLRP3-PANoptosome activity (49), miR-155 indirectly promotes NF-κB pathway activation and PANoptosis-related gene expression by suppressing its inhibitor, TNFAIP3, in liver transplant IRI (50). However, direct evidence for the involvement of non-coding RNA and metabolic regulation of PANoptosis in urological diseases remains limited, their established regulation of apoptosis, pyroptosis, and necroptosis in conditions such as renal cell carcinoma and diabetic nephropathy (DN) suggests a high potential for their involvement in urological PANoptosis, warranting further investigations.

Organelle dysfunction and metabolic stress are central triggers of PANoptosis, with mitochondrial defects, such as mtDNA leakage and mitochondrial ROS (mtROS) bursts, being key mediators. In IRI, lactic acid induces histone H3K18 lactylation, upregulates Arg1 expression, damages mitochondrial cristae and releases mtDNA, thereby activating ZBP1 (51, 52). In atrazine (ATR)-induced nephrotoxicity, ATR disrupts mitochondrial membrane integrity, leading to mtDNA release into the cytosol via mPTP and BAX pores. This activates the cGAS–STING pathway, triggering renal PANoptosis and inflammation. Lycopene (LYC) stabilizes mitochondrial membranes and inhibits this process (53). Endoplasmic reticulum and Golgi apparatus stress synergize with mitochondrial stress in pathological conditions, such as severe acute pancreatitis, collectively promoting ZBP1-dependent PANoptosis.

**Figure 1:**
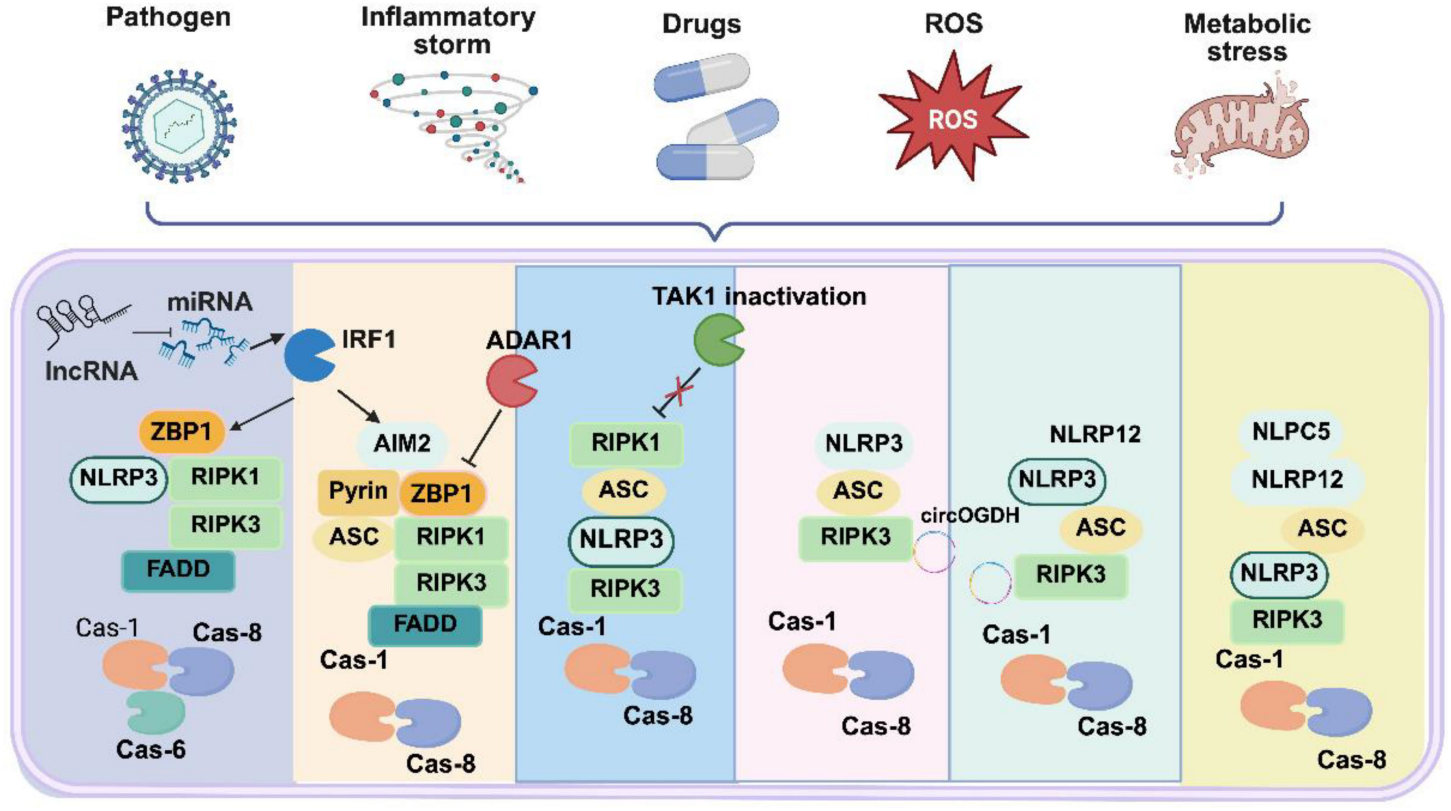
Molecular architecture of the PANoptosome and its regulatory network in urological diseases. The PANoptosome complex consists of a sensor layer (ZBP1, AIM2, NLRP3), an adapter layer (ASC, FADD), and an effector layer that concurrently activates apoptosis, pyroptosis, and necroptosis. The diagram also illustrates key upstream regulators, including transcriptional control by IRF1, metabolic stress, and non-coding RNAs, providing a framework for understanding PANoptosis initiation in urological diseases.

The three classic cell death pathways are spatiotemporally coordinated within the PANoptosome rather than following a strict sequence. This integrated assembly functions as a molecular hub that licenses potent positive feedback loops, such as caspase-8-mediated crossover activation of pyroptosis and necroptosis effectors, and mitochondrial DAMP-driven amplification of ZBP1 signaling—ensuring rapid and irreversible inflammatory cell death. Collectively, these findings illustrate that PANoptosis is a highly integrated and tightly regulated process, with diverse molecular checkpoints and modulators. Understanding its regulation advances cell death biology and provides a foundation for exploring its specific roles in urological diseases (54). The core molecular architecture of the PANoptosome and its regulatory network are summarized in **Figure 1**.

### Dual roles of PANoptosis in disease pathogenesis

**Figure 2:**
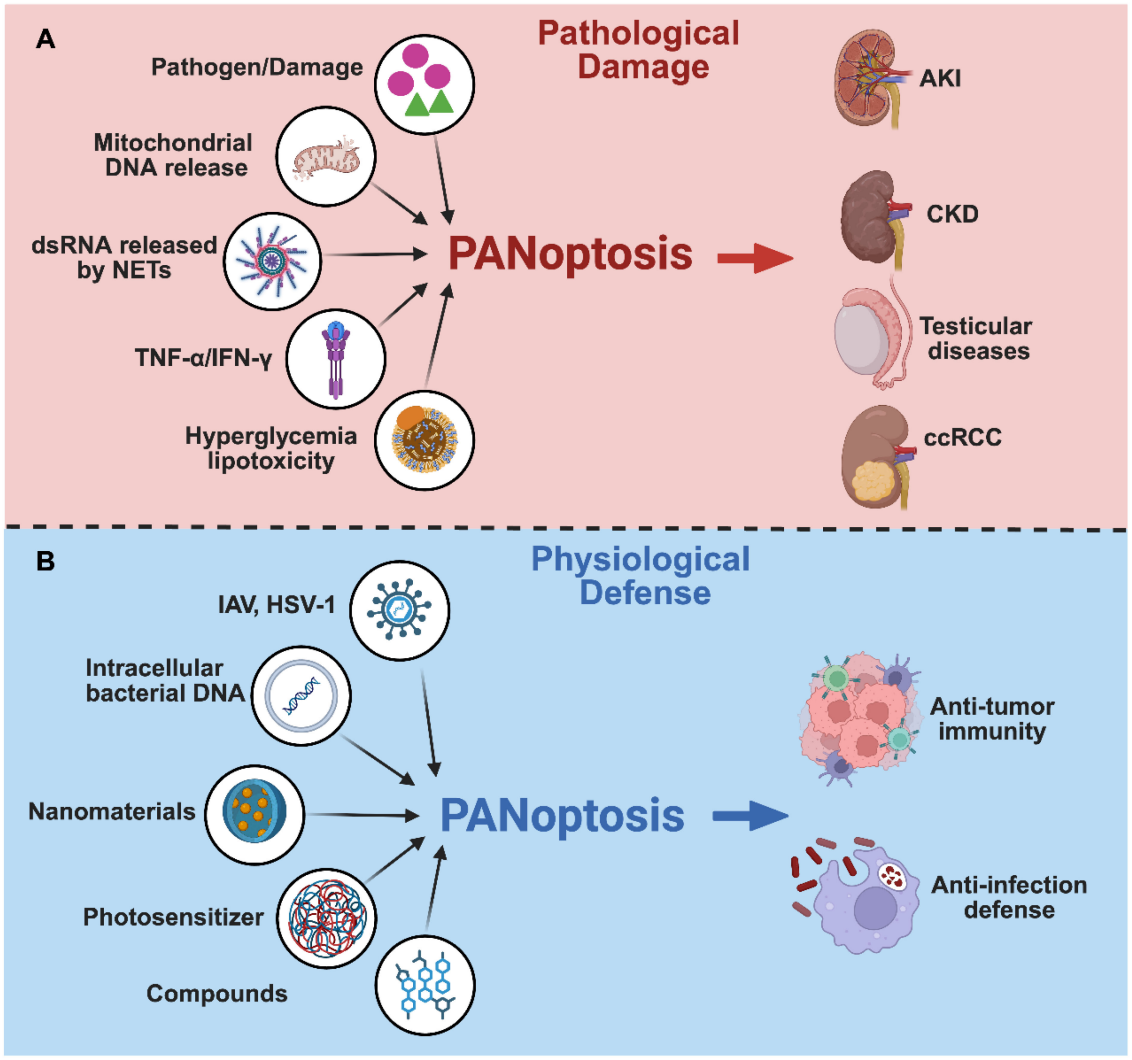
The dual role of PANoptosis in urological health and disease. **(A)** Pathological Damage: Triggers such as PAMPs/DAMPs, mitochondrial stress, and cytokines induce PANoptosis, contributing to AKI, CKD, testicular dysfunction, and renal carcinoma progression. **(B)** Physiological Defense: Pathogen-derived components or therapeutic agents trigger PANoptosis in infected or malignant cells, promoting anti-pathogen immunity and anti-tumor responses via immunogenic cell death.

PANoptosis plays a dual role in disease progression: it functions as a host defense mechanism to eliminate pathogens or malignant cells; however, its overactivation can cause irreversible tissue damage. This duality arises from the inflammatory amplification effect which integrates multiple cell death pathways. PANoptosis exerts a context-dependent, dual role in disease pathogenesis, as schematically illustrated in **Figure 2**.

PANoptosis is as a central driver of tissue injury and in disease exacerbation across various organ systems. This process is critically involved in the context of kidney diseases, such as AKI and Chronic Kidney Disease (CKD).

In metabolic diseases, high glucose levels activate podocyte PANoptosis via the TNF-α/TRAIL-DR5 axis, disrupting the glomerular filtration barrier. This involves the recruitment of FADD–caspase-8 and co-activation of caspase-3, GSDME, and RIPK3–MLKL (33). In diabetic cardiomyopathy, high glucose–induced mitochondrial ROS activates the circOGDH/HMGB1/RIPK3 axis in cardiomyocytes, leading to the activation of caspase-3, GSDMD, and p-MLKL, ultimately leading to heart failure (48). In nonalcoholic fatty liver disease (NAFLD), lipotoxicity triggers mtDNA release through the ER stress–mitochondrial dysfunction cascade, the activating the cGAS-STING pathway to upregulate ZBP1. This promotes PANoptosome assembly and activation of caspase-8 and RIPK3, driving hepatocyte death and fibrosis (55). In IRI, neuronal mtDNA activates ZBP1 in the central nervous system, driving RIPK3–caspase-8–GSDMD triple activation and accelerating neuronal lysis (56–58). In liver and kidney IRI, Kupffer cells upregulate ZBP1 via the TLR4-NF-κB axis to detect Z-DNA, whereas renal tubular epithelial cells recognize double-stranded RNA (dsRNA) released by neutrophil extracellular traps (NETs) via TLR3, activating parallel pathways—ZBP1–RIPK3–MLKL and caspase-1–GSDMD—leading to acute injury (59, 60). In autoimmune and inflammatory diseases, intestinal epithelial cells in patients with IBD upregulate the IRF1-ZBP1 module under IFN-γ stimulation, promoting RIPK3/caspase-8 oligomerization and co-localization of apoptosis, pyroptosis, and necroptosis markers, disrupting the tight junction protein ZO-1 (40, 41). In septic multi-organ failure, endotoxins and heme activates cGAS-STING–dependent PANoptosis via the ZBP1-PLCγ-mtDNA axis, with macrophages exhibiting GSDMD pores, Mixed Lineage Kinase Domain-Like Protein (MLKL) membrane translocation, and Terminal deoxynucleotidyl Transferase Mediated Nick End Labeling (TUNEL) positivity, which directly contributes to alveolar collapse (61).

In contrast, PANoptosis plays a protective role in immune surveillance and malignant cell clearance. In antitumor immunity, nanomaterials such as fullerol MF induce endoplasmic reticulum stress by disrupting lysosomal membranes and activating the caspase-8–PANoptosome switch. This results in high tumor cell expression of GSDME, cleaved caspase-3, and p-MLKL, along with the release of DAMPs including HMGB1 and ATP, which promote dendritic cell maturation and CD8^+^ T cell infiltration (62, 63). The sonosensitizer Mn-substituted HfO2 with a 20% Mn ratio (HMO) induces PANoptosis via the oxidative stress–ZBP1 axis, enhancing tumor sensitivity to PD-1 inhibitors (64). In glioma, the compound cinobufagin induces PANoptosis, promoting M1 polarization of tumor-associated macrophages and increasing T cell infiltration. This effect depends on mtDNA released from dying tumor cells, which is sensed by macrophage ZBP1 and activates the cGAS-STING-IFN-β pathway (44). During IAV infection, ZBP1 detects viral nucleic acids and activates the RIPK3–caspase-8–GSDMD cascade, inducing lysis of infected cells to restrict viral spread (17, 65). In bacterial pneumonia, AIM2 detects intracellular bacterial DNA and forms a complex with ASC–caspase-8, activating caspase-1/GSDMD and caspase-3 to clear infected cells while preventing excessive inflammation (2). *Mycobacterium tuberculosis* secretes effector proteins that inhibit RIPK1 phosphorylation and block PANoptosis, underscoring its role as a host defense checkpoint (2).

The balance between the pathological and protective functions of PANoptosis is influenced by: tissue-specific molecular preferences, microenvironmental signaling differences, and temporal feedback dynamics. In metabolic tissues such as the liver and kidney, the mitochondrial stress–ZBP1 axis predominantly mediates tissue-damaging effects. In contrast in immune cells, such as macrophages and T cells, the caspase‐8–GSDMD module facilitates pathogen clearance (2, 36, 51, 55, 59). In the tumor microenvironment, low pH and high ROS promote RIPK1–caspase-8 positive feedback, enhancing immunogenic cell death, whereas TGF-β in fibrotic environments suppresses AIM2 expression via Smad3, reducing the antifibrotic effects of PANoptosis (62, 63, 66). Early infection induces ZBP1-dependent PANoptosis for rapid pathogen clearance (within 6 h of IAV infection); however, prolonged inflammation triggers IFN–γ–IRF1–ZBP1 feedback, leading to tissue damage, as observed in cases of septic lung failure (17, 38, 65).

## Role and molecular mechanisms of PANoptosis in urological diseases

### Acute kidney injury

In AKI, PANoptosis, a synergistic form of programmed cell death, contributes to renal pathology through multiple molecular mechanisms triggered by diverse stimuli. In sepsis-associated AKI, renal AIM2 expression is markedly upregulated, accompanied by GSDMD cleavage (pyroptosis), caspase-3 activation (apoptosis), and p-MLKL accumulation (necroptosis). EIF2AK2 directly binds to AIM2 to drive this process, whereas the AIM2 inhibitor a151 effectively attenuates renal injury (22). Viral sepsis, such as H1N1 infection, can spread hematogenously to the kidneys, inducing endothelial PANoptosis and leading to interstitial inflammation and vascular abnormalities (65).

In IRI models, dsRNA released by NETs activates tubular epithelial PANoptosis through TLR3. PAD4 knockout or TLR3 inhibition alleviates this injury (60). Similarly, the single-atom enzyme Pt/SAE reduces PANoptosis by scavenging ROS, blocking Z-DNA formation, and suppressing ferroptosis (67). Drug- and toxin-induced AKI involves PANoptosis. Aristolochic acid (AAI) upregulates histone deacetylase (HDAC) 1/2 to repress PSTPIP2 expression, thereby inducing tubular PANoptosis, which can be reversed by the HDAC inhibitor romidepsin or PSTPIP2 overexpression (68). Similarly, cadmium exposure (5–15 μM CdCl_2_) triggers PANoptosis in macrophages through caspase-1/GSDMD activation, caspase-3 cleavage, and MLKL phosphorylation, potentially amplifying tubular inflammatory death (69).

### Chronic kidney disease

PANoptosis plays a central role in CKD. Single-cell sequencing has demonstrated the activation of the TRAIL/DR5 pathway in podocytes from patients with DN, which simultaneously induces apoptosis, pyroptosis, and necroptosis. Podocyte-specific knockout of TNFSF10 or TNFRSF10B markedly reduces this injury (33). Bioinformatic analyses have identified PANoptosis-related gene signatures (PDK4, YWHAH, and PRKX) that are aberrantly expressed in DN and associated with immune microenvironment dysregulation, particularly M2 macrophage infiltration (70). Additionally, FOS and PTGS2 act as hub genes linking PANoptosis to inflammation and fibrosis in CKD (71). Methylation quantitative trait locus (mQTL) analysis further associates altered methylation of CCND1, HGF, and MADD with CKD risk (72). External factors can aggravate CKD pathology: for instance, burns aggravate renal injury in mouse models by promoting M1 macrophage polarization and caspase-1/3-dependent PANoptosis (73). The herbicide ATR induces PANoptosis by promoting mtDNA release and activating the cGAS-STING pathway, whereas LYC protects against this process by stabilizing mitochondrial membranes (53).

### Testicular diseases

In Wilson’s disease, abnormal copper accumulation activates the TLR4/NF-κB signaling pathway, inducing PANoptosis in the testicular tissue. This process is characterized by NLRP3 inflammasome activation, caspase-3 cleavage, and MLKL phosphorylation, ultimately leading to spermatogenic cell death and reduced sperm count. Treatment with the copper chelator penicillamine or the TLR4 inhibitor Eritoran significantly inhibits PANoptosis and improves spermatogenic function (74). Although direct evidence linking other toxicants to testicular PANoptosis is limited, existing studies suggest their possible reproductive toxicity. Maternal exposure to triclosan (TCS) induces lung fibrosis in offspring, accompanied by increased PANoptosis markers, highlighting the potential risks to reproductive health.

### Renal tumors

In clear cell renal cell carcinoma (ccRCC), PANoptosis promotes tumor progression through multiple molecular mechanisms. Genes such as CASP4, TLR3, CASP5, and PYCARD are markedly overexpressed in ccRCC, driving cell death via activation of signaling axes including BAX–Bcl-xL–caspase-3–GSDME and ZBP1–RIPK1–RIPK3–MLKL (75–77). Elevated PANoptotic activity correlates with advanced tumor stage (III/IV), higher pathological grade (G3/G4), and poor prognosis (75, 78). These effects are linked to an immunosuppressive microenvironment characterized by increased M2 macrophage infiltration, enrichment of regulatory T cells (Tregs), and reduced populations of CD4^+^ T and natural killer (NK) cells (77, 79). Several prognostic models have been developed to evaluate the clinical relevance of PANoptosis. A three-gene model based on CASP4, LY96, and TLR3 (risk score = 0.5787 × CASP4 + 0.1402 × LY96 – 0.4056 × TLR3) identified a high-risk group with frequent BAP1 mutations, advanced disease, and reduced 5-year survival rate (77, 79). Another five-gene miRNA model based on miR-200a-5p, miR-21-5p, and miR-223-3p showed that the low-risk group was enriched in fatty acid metabolism pathways, whereas the high-risk group was enriched in inflammation-related pathways (75). Long noncoding RNAs (lncRNAs) such as LINC00944, LINC02611, and PRKAR1B-AS1 are positively associated with CD8^+^ T cell infiltration and immune checkpoint expression, providing additional risk-stratification markers (80).

Therapeutically, tumors with high PANoptosis scores exhibit greater tumor mutation burden (TMB), enhanced infiltration of CD8^+^ T and dendritic cells, and higher objective response rates (ORR) to PD-1/CTLA-4 inhibitors (42% *vs* 18%) (76, 79). The high-risk group is more sensitive to paclitaxel, whereas the low-risk group responded better to sunitinib (79). Experimental studies have demonstrated that CASP5 knockdown reduces proliferation, migration, and tumor formation in 786-O cells (77). Conversely, PYCARD overexpression promotes M1 macrophage polarization, and its binding to CASP1 is inhibited by the small-molecule compound oroxin B (76).

In other renal cancer subtypes such as kidney papillary renal cell carcinoma (KIRP) and kidney chromophobe renal cell carcinoma (KICH), the PANoptosis immune index (PANII) was constructed using BAX, CASP1, CASP8, and PYCARD. High PANII scores correlated with increased TMB, upregulation of immune checkpoint molecules, and improved immunotherapy response (76). Although PANoptosis pathway enrichment was lower in KIRP and KICH than in ccRCC, it remained associated with immune cell infiltration and patient prognosis (76).

### Prostate tumors

PANoptosis exerts tumor-suppressive effects in prostate cancer (PRAD). A high PANoptosis score—such as that from a four-gene model including CASP7, ADAR, DNM1L, and NAIP—was associated with longer overall survival (HR = 0.36, p < 0.001) and higher immunotherapy response rates (81, 82). These effects reflect “hot tumor” features, including increased CD8^+^ T cell infiltration, upregulation of immune checkpoint molecules (PD-1 and CTLA-4), and activation of the IFN-γ pathway (81, 82). The four-gene model (risk score = 0.9075 × CASP7 + 0.0645 × ADAR + 0.6644 × DNM1L + 1.1522 × NAIP) stratified patients into high- and low-risk groups (82). The high-risk group exhibited lower tumor purity, reduced Treg cells, and a greater risk of biochemical recurrence (81).

Patients with a high PANII score had lower TIDE scores, indicating an improved immunotherapy response. Functional studies have demonstrated that ADAR silencing inhibits PC-3M cell proliferation, reverses epithelial–mesenchymal transition (EMT), increases sensitivity to docetaxel, and induces M1 macrophage polarization (82). Additionally, arsenic (NaAsO_2_) activates PANoptosis in prostate cells via mitochondrial damage, whereas phenyllactic acid (PLA) suppresses Casp8 activity through the NFS1/eIF4B/WTAP axis, promoting chemotherapy resistance (83, 84). Clinical analyses have showed that serum PLA levels are lower in patients with prostate cancer than in those with benign prostatic hyperplasia or prostatitis and are negatively correlated with the Gleason score (84). PANoptosis exhibited opposite effects in ccRCC and PRAD, which reflects its biological nature as a “dual role”, and closely related to the genetic background and microenvironment of the tumor. As detailed above, PANoptosis contributes to a wide spectrum of urological pathologies, with key inducers, sensors, effectors, and functional outcomes summarized in **Table 1**.

**Table 1:** Pathophysiological roles and evidence of PANoptosis in urological disorders.

Disease	Inducer/model	Key sensors/pathways	Effectors activated	Functional outcome & clinical correlation	Refs.
AKI	Sepsis	EIF2AK2 directly binds and drives AIM2 inflammasome activation	Caspase-1, GSDMD, Caspase-3, p-MLKL	Renal tubular injury; ameliorated by AIM2 inhibitor a151	(22)
	Ischemia-Reperfusion Injury (IRI)	NETs-derived dsRNA activates ZBP1 via the TLR3 pathway	RIPK3, MLKL, Caspase-1, GSDMD	Renal tubular epithelial cell death; alleviated by PAD4 knockout or TLR3 inhibition	(60)
	Aristolochic Acid I (AAI)	HDAC1/2 upregulation leads to transcriptional suppression of PSTPIP2	/	Renal tubular PANoptosis; reversible by HDAC inhibitor Romidepsin	(68)
	Cadmium (Cd) Exposure	/	Caspase-1, GSDMD, Caspase-3, p-MLKL	Macrophage PANoptosis, driving renal tubular inflammation	(69)
CKD	Diabetic Nephropathy (DN)	TRAIL/DR5 Pathway	Caspase-3, GSDME, RIPK3-MLKL	Podocyte death, filtration barrier disruption; alleviated by TNFSF10/TNFRSF10B knockout	(33)
	Diabetic Nephropathy (DN)	/	PDK4, YWHAH, PRKX (Gene Signature)	Associated with M2 macrophage infiltration and immune microenvironment dysregulation	(70)
	Atrazine (ATR)	mtDNA release and subsequent activation of the cGAS-STING pathway	/	Renal PANoptosis; lycopene ameliorates by stabilizing mitochondria	(53)
Testicular Disease	Wilson’s Disease (Copper Accumulation)	Cu^2^ activates the TLR4/NF-κB signaling pathway	NLRP3, Caspase-3, p-MLKL	Spermatogenic cell death, reduced sperm count; improved by copper chelators or TLR4 inhibitors	(74)
ccRCC	Tumor Progression	ZBP1/RIPK1/RIPK3/MLKL or BAX/Caspase-3/GSDME	Caspase-3, GSDME, p-MLKL	Pro-tumorigenic: Associated with advanced stage, higher grade, poor prognosis, and immunosuppressive microenvironment (increased M2 macrophages, Tregs)	(75, 77–79)
PRAD	Tumor Immune Surveillance	Model based on CASP7, ADAR, DNM1L, NAIP	/	Anti-tumorigenic: Correlated with longer overall survival, “hot” tumor microenvironment (CD8^+^ T cell infiltration, upregulated immune checkpoints), and higher response rate to immunotherapy	(81, 82)

TLR4, Toll-like Receptor 4; NLRP3, NACHT; LRR and PYD domains-containing protein 3; p-MLKL, phosphorylated Mixed Lineage Kinase domain-Like protein.

CASP7, Caspase 7; ADAR, Adenosine Deaminase Acting on RNA; DNM1L, Dynamin-1-like protein; NAIP, Neuronal Apoptosis Inhibitory Protein.

### Anti-tumor therapy

The PANoptosis status has important predictive value for immunotherapy and chemotherapy response. High PANoptosis scores are positively correlated with TMB and immune cell infiltration, suggesting improved responses to immune checkpoint inhibitors (anti-CTLA-4/PD-1 therapy) (76, 79, 81, 82). Patients with low TIDE scores and high immune phenotype scores (IPS) were more likely to benefit from immunotherapy (81, 82). PANoptosis status influences drug sensitivity to chemotherapy and targeted therapy. In ccRCC, the high-risk group was sensitive to paclitaxel, whereas the low-risk group responded better to sunitinib (79). Certain compounds, including arsenic and CBL0137, induce PANoptosis and thereby enhance anticancer efficacy; however, caution is warranted because of the risk of systemic inflammatory response (83, 85).

## Therapeutic targets and strategies for PANoptosis in the urinary system

**Figure 3:**
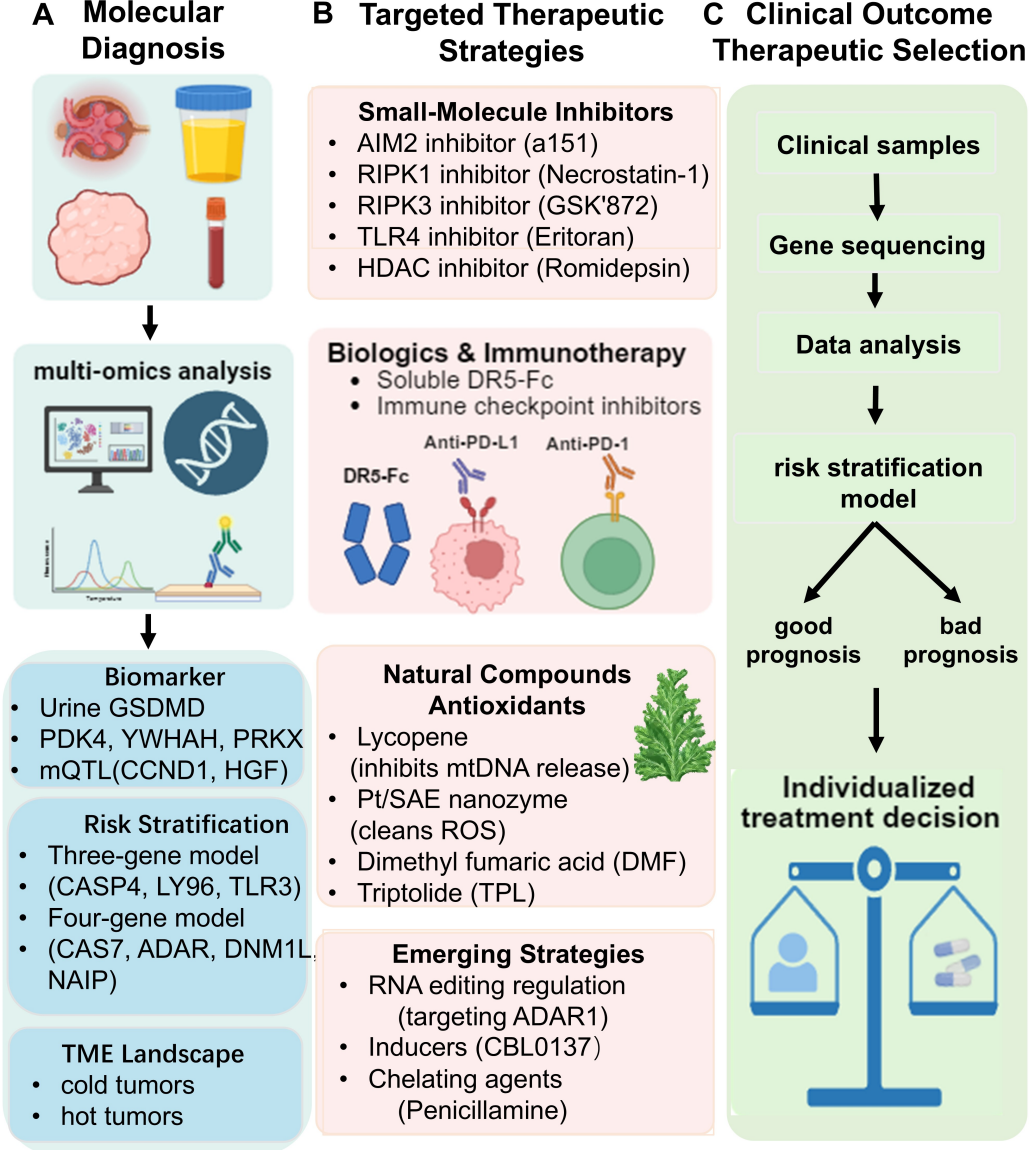
Schematic overview of the translational roadmap for PANoptosis-targeted precision medicine in urological diseases. The schematic outlines a strategy for implementing PANoptosis-based diagnostics and therapies: **(A)** Molecular Diagnosis utilizing multi-omics and liquid biopsies; **(B)** argeted Therapeutic Strategies encompassing small-molecule inhibitors, biologics, natural compounds, and emerging agents; and **(C)** Clinical Outcome & Therapeutic Selection guided by biomarkers, risk stratification, and the tumor microenvironment to achieve precision medicine in urology.

Therapeutic strategies for PANoptosis in the urinary system include targeting key signaling molecules, blocking execution pathways, and controlling upstream inducers. These approaches are characterized by multitarget and multipathway interventions. A translational roadmap for targeting PANoptosis in urological diseases, encompassing diagnostic stratification and therapeutic intervention, is provided in **Figure 3**; **Table 2**.

**Table 2:** Therapeutic strategies targeting the PANoptosis pathway.

Strategy	Agent/compound	Target/mechanism	Tested model/disease	Outcome	Refs.
Targeting Sensors	AIM2 inhibitor (A151)	Inhibits AIM2 inflammasome assembly	Sepsis-associated AKI	Attenuates renal tubular PANoptosis and renal impairment	(22)
	Eritoran	TLR4 antagonist	Copper-induced testicular injury	Suppresses PANoptosis and improves spermatogenic function	(74)
	(siRNA/inhibitor)	Targets IRF1	Endothelial cell PANoptosis	Reduces cell death; potential for glomerular diseases/transplant rejection	(32)
Inhibiting Adapters/Kinases	Necrostatin-1 (Nec-1)	RIPK1 inhibitor	Renal transplantation IRI model	Concurrently inhibits apoptosis, pyroptosis, and necroptosis; alleviates injury	(86)
	GSK’872	RIPK3 inhibitor	Combined with IDN-6556	Nearly completely suppresses TPL-induced macrophage PANoptosis	(87)
Inhibiting Effectors	IDN-6556	Pan-caspase inhibitor	Combined with GSK’872	Nearly completely suppresses TPL-induced macrophage PANoptosis	(87)
	sDR5-Fc	Neutralizes TRAIL/DR5 signaling	Diabetic nephropathy model	Inhibits podocyte PANoptosis and exerts protective effects	(33)
Controlling Upstream Triggers	Pt/SAE nanozyme	Scavenges ROS; inhibits Z-DNA formation and ferroptosis	Renal IRI	Significantly ameliorates ZBP1-mediated PANoptosis	(67)
	Lycopene	Inhibits mtDNA release and cGAS-STING pathway activation	ATR-induced kidney injury	Alleviates renal PANoptosis and inflammatory response	(53)
	Dimethyl fumarate (DMF)	Inhibits mtROS production and PANoptosome assembly	Kidney injury model	Significantly mitigates renal injury	(88)
	Penicillamine	Copper chelator	Wilson’s disease-related testicular injury	Reduces copper deposition; inhibits TLR4/NF-κB pathway; protects spermatogenic cells	(74)
Epigenetic Regulation	Romidepsin	HDAC inhibitor; upregulates PSTPIP2 and inhibits Caspase-8	AAI-induced kidney injury	Significantly reduces renal PANoptosis	(68)
Immunomodulation	Anti-PD-1/CTLA-4	Immune checkpoint inhibitors	Cancers with high PANoptosis score (renal/prostate)	Higher objective response rate (42% *vs* 18%)	(79, 81, 85)
	CBL0137	Induces Z-DNA formation and PANoptosome assembly	macrophages	Induces PANoptosis; potential for combination immunotherapy to reverse “cold” tumors	(85)
Natural Products	*Achyranthes bidentata* water extract (AAW)	Activates AMPK/mTOR; promotes autophagy; inhibits PANoptosis	Cisplatin-induced AKI	Significantly improves renal injury	(89)
	Triptolide (TPL)	(Specific target not fully elucidated)	Macrophage model	Induces PANoptosis, which can be blocked by inhibitor combination	(87)

AKI, Acute Kidney Injury; IRI, Ischemia-Reperfusion Injury; AAI, Aristolochic Acid I; mtROS, Mitochondrial Reactive Oxygen Species; TLR4, Toll-like Receptor 4; IRF1, Interferon Regulatory Factor 1; RIPK1/3, Receptor-Interacting Serine/Threonine-Protein Kinase 1/3; TRAIL/DR5, TNF-Related Apoptosis-Inducing Ligand/Death Receptor 5; ZBP1, Z-DNA Binding Protein 1; cGAS-STING, Cyclic GMP-AMP Synthase - Stimulator of Interferon Genes; HDAC, Histone Deacetylase; PSTPIP2, Proline-Serine-Threonine Phosphatase-Interacting Protein 2; PD-1/CTLA-4, Programmed Cell Death Protein 1/Cytotoxic T-Lymphocyte-Associated Protein 4; AMPK/mTOR, AMP-Activated Protein Kinase/Mechanistic Target of Rapamycin.

### Targeting key signaling molecules

ZBP1 is a core sensor molecule in PANoptosome formation and plays an essential role in kidney injury. Studies have shown that during viral infection or IFN-β stimulation, Zbp1 deletion completely blocks renal PANoptosis and significantly improves survival (16). Small-molecule inhibitors or small interfering RNAs (siRNA) targeting ZBP1–ZNA interactions have emerged as potential therapies for viral or inflammatory kidney injuries. The EIF2AK2–AIM2 axis is strongly activated in sepsis-induced AKI. The inhibitor a151 effectively suppresses AIM2 inflammasome assembly, decreases caspase-1 activity and GSDMD cleavage, and thereby alleviates tubular PANoptosis and kidney dysfunction (22). IRF1 has been identified as a key regulator of TNFα/IFNγ-induced endothelial PANoptosis (32). siRNA or small-molecule inhibitors targeting IRF1 reduced cell death, highlighting their therapeutic potential in glomerular disease and kidney transplant rejection. In heavy metal–induced testicular or kidney damage (copper, cadmium), the TLR4/NF-κB pathway is activated. TLR4 inhibitors such as Eritoran or natural compounds like lycopene, attenuate PANoptosis and consequent tissue damage by suppressing this pathway (74).

### Intervening in cell death execution mechanisms

Studies have indicated that combined inhibition of multiple cell death pathways is more effective than a single inhibition. Necrostatin-1 (Nec-1), an RIPK1 inhibitor, simultaneously inhibits apoptosis, pyroptosis, and necroptosis in a rat kidney transplantation model, reducing IRI (86). Triptolide (TPL)-induced macrophage PANoptosis can be almost completely blocked by co-treatment with IDN-6556 (a pan-caspase inhibitor) and GSK’872 (a RIPK3 inhibitor) (87). NS (3, 4-methylenedioxy-β-nitrostyrene) inhibits NLRP3 inflammasome assembly, decreasing caspase-1 activation and IL-1β release, thereby alleviating kidney PANoptosis during IRI (24). The HDAC inhibitor romidepsin reduced the severity of AAI-induced kidney PANoptosis by upregulating PSTPIP2 expression and suppressing caspase-8 activity, offering a therapeutic approach for toxic kidney injury (68).

### Controlling upstream inducers and antioxidant strategies

Mitochondrial reverse electron transport (RET) is a major upstream inducer of PANoptosis. Dimethyl fumarate (50 mg·kg^-1^ d^-1^) protects the kidneys by inhibiting mtROS production and PANoptosome assembly (88). Pt/SAE nanozymes mimic the activity of superoxide dismutase and catalase, scavenge ROS, inhibit ferroptosis, and reduce Z-DNA formation, thereby limiting ZBP1-mediated PANoptosis in IRI (67). LYC attenuates kidney PANoptosis and inflammation by blocking ATR-induced mitochondrial DNA release and cGAS-STING pathway activation (53). In diabetic kidney disease (DKD), targeting TRAIL/DR5 signaling can inhibit podocyte PANoptosis, with soluble DR5-Fc showing therapeutic effects in animal models (33). In cadmium or copper-induced testicular PANoptosis, chelators such as penicillamine reduce metal ion deposition, inhibit TLR4/NF-κB pathway activation, and protect spermatogenic and Sertoli cells (74). Recent studies have shown that PLA inhibits PANoptosis in prostate cancer cells by upregulating NFS1 and promoting SUMOylation of TNF-α, suggesting that metabolic intervention may represent a therapeutic strategy (84).

### Immunomodulation and RNA editing strategies

Several studies have developed PANoptosis-related risk models based on lncRNAs or gene expression (76, 77, 79, 80, 82). These models showed that high-risk scores were associated with immunosuppressive microenvironments, upregulation of immune checkpoint molecules (PD-1, CTLA-4, and LAG3), and increased immune cell infiltration. This suggests that patients with high PANoptosis scores may be more responsive to immune checkpoint inhibitor (ICI) therapy. In kidney cancer, patients in the high-risk group may benefit more from combined anti–PD-1/CTLA-4 therapy (77, 80). Induction of tumor cell PANoptosis can be achieved using CBL0137, a small molecule compound that promotes Z-DNA formation, drives PANoptosome assembly, and induces PANoptosis in macrophages *in vitro (*85). Although its combination with LPS in mice exacerbates multi-organ injury, and this potent induction capacity suggests that pairing it with immunotherapy could selectively trigger PANoptosis in tumor cells, thereby activating antitumor immunity and converting a “cold” tumor microenvironment into a “hot” one (85).

Emerging RNA editing strategies have attracted attention. ADAR1-p150 inhibits PANoptosis and inflammatory responses by competitively binding Z-RNA to ZBP1. ADAR1-dependent A-to-I RNA editing alleviates PANoptosis; however, ADAR overexpression may cause off-target editing throughout the transcriptome. Therefore, strategies that recruit endogenous ADAR, rather than overexpressing it (CRISPR/dCas13-based systems), may offer greater therapeutic efficacy and safety.

### Traditional Chinese medicine and natural product therapy

The water extract of *Achyranthes bidentata* (AAW) improves cisplatin-induced AKI by activating pro-survival signaling pathways such as AMPK/mTOR, promoting autophagy and mitophagy, and suppressing apoptosis, necroptosis and PANoptosis (89). Natural compounds, including TPL and LYC also modulate PANoptosis through distinct mechanisms, highlighting their therapeutic potential (53, 87).

In summary, PANoptosis—an inflammatory death pattern that integrate multiple programmed cell death pathways—plays a central role in various urological diseases, including AKI, CKD, renal carcinoma, and prostate disorders. The molecular mechanisms of pyroptosis, which involve the activation of key sensors such as ZBP1, AIM2, and IRF1, the dynamic assembly of PANoptosomes, and the fine-tuned regulation of non-coding RNAs and metabolic reprogramming, deepen our understanding of disease progression and offer novel diagnostic, prognostic, and therapeutic perspectives for urological clinical practice. A molecular classification system based on PANoptosis-associated gene signatures, immune microenvironment characteristics, and liquid biopsy markers holds promise for early disease identification and risk stratification. Inhibitors, natural compounds, and nanodelivery systems targeting key nodes such as ZBP1, caspase-8, and RIPK1 demonstrate significant therapeutic potential for multi-targeted intervention and synergistic blockade of inflammatory cell death. In the future, integrating single-cell multi-omics, spatial transcriptomics, and AI predictive models to advance the clinical translation of PANoptosis mechanisms will establish a novel paradigm for achieving precision diagnosis, treatment, and personalized therapy for urological diseases.

### Limitations and future perspectives

Despite significant progress in PANoptosis research on urinary system diseases, clinical translation faces major challenges. Most mechanistic studies rely on animal models or cell lines and have limited validation in human tissues. In intrahepatic cholangiocarcinoma (ICC), mouse models have demonstrated that ZBP1 deficiency suppresses PANoptosis. However, clinical sample analysis indicates that CAF-derived POSTN attenuates the ZBP1 effect by recruiting TAMs (90). This disconnect has led to most intervention strategies remaining confined to the preclinical stage.

Disease heterogeneity is often overlooked, and existing predictive models may not apply to all patients (91, 92). The critical dynamic processes of PANoptosome assembly, such as the timing of RIPK1/RIPK3 activation in renal tubular injury, remains unknown (34). The nucleic acid threshold for ZBP1 activation (the level of mtDNA leakage required to trigger complex assembly in UV models) remains unknown (18).

Current detection methods rely on tissue biopsies (immunohistochemical analysis of p-MLKL/GSDMD colocalization), which preclude dynamic monitoring. The exploration of alternative biomarkers in blood or bodily fluids (mtDNA fragments, ZBP1-containing extracellular vesicles) remains in its infancy (93, 94). PANoptosis exhibits tissue- and cell type-specific differences proximal and distal tubular epithelial cells display distinct death patterns in renal ischemia-reperfusion injury, highlighting the need for therapies targeting specific cell types (20, 60).

Safety and targeting remain major hurdles sDR5-Fc may impair immune surveillance, and existing nanocarrier-based delivery systems show limited efficiency in tissues (33, 54, 95). Research is constrained by sample heterogeneity, as most studies use retrospective data without prospective multicenter validation (17, 43, 56). Mechanistic depth is insufficient, and the interplay between PANoptosis, metabolic reprogramming, and epigenetic regulation (m6A modification) remains unclear (43, 96).

Future research should prioritize multi-omics and biomarker development by combining single-cell and spatial transcriptomic technologies to identify urinary tissue–specific biomarkers (phosphorylated ZBP1) and noninvasive diagnostic indicators in body fluids (urinary GSDMD fragments) (55). By constructing disease-specific organoids, we simulated the human microenvironment to evaluate drug responses.

Technologies for dynamic mechanism analysis, such as real-time imaging tools (FRET probes) to track PANoptosome assembly processes and computational models that predict optimal intervention windows, are essential (34). Establish PANoptosome kinetic models (RIPK1 phosphorylation energy barrier calculations) to predict intervention targets (therapeutic window for PGAM5 inhibitors in subarachnoid hemorrhage) (34).

Cell-specific therapeutic strategies, including targeted delivery systems (antibody-conjugated nucleic acids targeting renal podocytes) and conditional gene editing to regulate ZBP1 expression, should be explored (33, 97).

Combination therapies and smart materials, such as sequential interventions (enhancing ZBP1 early during infection and inhibiting RIPK1 later) or microenvironment-responsive materials (such as pH-triggered drug-release hydrogels) are promising (17, 38, 67). Additionally, combination therapies (such as CBL0137 combined with immune checkpoint inhibitors) should be developed, guided by single-cell analyses (scRNA-seq to reveal LINC00944’s role in T-cell infiltration) and cross-cancer mechanistic comparisons to clarify the opposing roles of PANoptosis in prostate and kidney cancer (98–101).

Future research should examine the dynamic evolution of PANoptosis in chronic diseases (DN), advance targeted delivery systems (e.g., nanoenzymes) for precision therapy, and validate the clinical value of ZBP1/p-MLKL as renal biopsy markers.
